# Supplementary material for: Thyrotoxicosis in a Postpartum Adolescent: A Simulation Case for Emergency Medicine Providers
Source: MedEdPORTAL. 2020 Sep 10;16:10967. doi: 10.15766/mep_2374-8265.10967 (PMC7485909; doi:10.15766/mep_2374-8265.10967)
Supplement: Supplementary file 1 — Thyroid Storm Simulation Case.docxSimulation Scenario Environment Checklist.docxThyroid Storm Case Labs - CXR, EKG & Photo.docxThyroid Storm Cardiac POCUS.mp4Thyroid Storm Lung POCUS.mp4Thyroid Storm IVC POCUS.mp4Thyroid Storm Debriefing Guide.docxThyroid Storm Debrief.pptxThyroid Storm Case Survey.docx [file mep_2374-8265.10967-s001.zip › A. Thyroid Storm Simulation Case.docx]

| **Appendix A: *MedEdPORTAL* Simulation Case**  **SIMULATION CASE TITLE:** Respiratory Distress and Altered Mental Status in a Post-Partum Adolescent  **AUTHORS**: Julie I Leviter, MD, Sakina Sojar, MD, Nina Ayala, MD & Robyn Wing, MD, MPH  **LEARNER AUDIENCE:** Emergency Medicine Residents, Emergency Medicine Advanced Practice Practitioners (APPs), Pediatric Emergency Medicine Fellows | |
| --- | --- |
| **PATIENT NAME:** Lily Brown  **PATIENT AGE:** 17 years old  **CHIEF COMPLAINT:** Respiratory distress and altered mental status  **PHYSICAL SETTING:** (Pediatric) Emergency Department | |
|  | |
| **Brief narrative description of case** | A 17-year-old adolescent female presents with respiratory distress and altered mental status occurring 2 days post-partum. Learner goals include to recognize, evaluate, and manage respiratory distress and altered mental status, recognize thyroid storm from history and physical findings, and mobilize the appropriate personnel and resources to manage an acutely ill post-partum patient. |
| **Primary Learning Objectives** | 1. Demonstrate recognition and management of respiratory distress and altered mental status in a post-partum patient. 2. Evaluate for causes of respiratory distress and altered mental status in a post-partum patient. 3. Recognize thyroid storm in a post-partum patient. 4. Manage thyroid storm in a post-partum patient. 5. Mobilize the appropriate personnel and resources to manage the peri-partum patient in the emergency department setting. 6. Demonstrate efficient and effective teamwork and communication skills. |
| **Critical Actions** | 1. Request intravenous access 2. Apply monitor and verbalize vital sign abnormalities of hyperthermia, tachycardia, tachypnea, and hypoxia 3. Perform a physical exam and verbalize the findings of goiter, agitation, sweating, respiratory distress, rales on pulmonary auscultation 4. Ask the patient’s family member what events led up to the current presentation, the patient’s medical and surgical history, allergies, medications, and family history. 5. Apply a nonrebreather mask either by asking the RN to do so, or by personally doing so, and start flow rate of at least 10L/min 6. Prepare intubation equipment including both direct and/or video assisted options 7. Request a chest radiograph, and verbalize the finding of pulmonary edema 8. Request an EKG, and verbalize the finding of atrial fibrillation with rapid ventricular response 9. Perform POCUS of heart, lungs, and IVC, and verbalize the findings of a hyperdynamic heart, pulmonary edema, and high output heart failure. 10. Order labs including, but not limited to, CBC, BMP, BNP, blood gas, TSH and TFTs, and verbalize the finding of an elevated BNP 11. Consult/discuss with maternal-fetal medicine the management of thyroid storm in a post-partum patient 12. Administer medical treatment including propylthiouracil (PTU) 300 mg – 1000 mg PO, propranolol 0.5 – 2mg IV or 20-80mg PO/NG, or esmolol 250 – 500 mcg/kg IV, dexamethasone 2mg IV or hydrocortisone 100mg IV^1^ 13. Admit to Intensive Care Unit |
| **Learner Preparation or Prework** | Assure learners of psychological safety during and after their participation. This brief simulation is intended to be formative and is solely for learning purposes.  The facilitators orient learners to the standardized patient, encourage them to treat her like a real live patient, and encourage learners to ask questions if they are unsure of something due to the simulation setting. Inform learners that they will be working as a team in caring for this patient.  The facilitators describe the setting for this scenario as follows:  Lily, a 17-year-old adolescent female, who is 2 days post-partum from a vaginal delivery, presents with trouble breathing and she seems confused. She is accompanied by her partner and/or parent (depending on actor availability). |

| Initial Presentation | | | | | |  |
| --- | --- | --- | --- | --- | --- | --- |
| **Initial vital signs** | | T 40°C  BP 110/75  HR 140  RR 24  O2 80% | | | | |
| **Overall Setting and Appearance** | | The patient appears agitated, anxious, and short of breath. She is asking inappropriate questions and speaking in short sentences with anxious/agitated tone. She can demonstrate agitation throughout the case by resisting monitor placement, trying to remove her oxygen mask, and attempting to get out of the bed. Examples of inappropriate questions and phrases include, but are not limited to, the following:   - - “Who are you?”   - “Where am I?”   - “Stop touching me!”   The patient is wearing a hijab which covers her neck area (alternatively could be a scarf or a turtleneck shirt). No monitors are on the patient.  Her partner and/or parent is available to answer questions. | | | | |
| **Confederates (e.g., standardized participants) and their roles in the room at case start** | | Nurse #1: The nurse is appropriately concerned and does not typically care for laboring or post-partum patients. The nurse is helpful and knowledgeable. However, only those interventions requested by the learner(s) should be performed. If learners do not identify thyromegaly on exam, the nurse may need to point it out. The nurse can pretend to first notice thyromegaly when readjusting the patient’s monitor leads or when placing leads for EKG.  Nurse #2: OPTIONAL ROLE. The nurse is appropriately concerned and does not typically care for laboring or post-partum patients. The nurse is helpful and knowledgeable. He/she may suggest calling a consultant for help.  Partner or Parent: Role can vary based on actor availability. Partner or parent is available to answer questions regarding the patient’s history and events leading up to current ED presentation. They are appropriately concerned. They redirect the agitated patient to keep oxygen on face and monitors including the oxygen saturation probe and cardiac leads appropriately placed.  Faculty instructor: Present, either in person, or in the control room for simulation equipment. He or she verbalizes physical exam findings (such as rales, irregular heart rhythm, etc.) upon observing the learners perform that aspect of the exam on the SP. This faculty instructor may serve as the voice of consultants including the maternal-fetal medicine or OB/GYN. The faculty instructor observes the performance of the learner(s), provides feedback and instruction to the nurse to facilitate case progression, and facilitates the debriefing session. | | | | |
| **HPI** | | Information volunteered by partner/parent:  Lily is a 17-year-old girl who is 2 days post-partum after an uncomplicated vaginal delivery. She believes she was close to full term and did have regular prenatal care with her midwife. After a normal spontaneous vaginal delivery two days ago, she was discharged from the hospital earlier today. This afternoon, she started having trouble breathing, asking strange questions and seeming agitated and anxious. So, her family brought her to the Emergency Department.  The following details will be revealed only when asked by the learner:   - She had palpitations and fatigue throughout her pregnancy - She had been told at the end of the pregnancy that she was hypertensive, but her urine tests were always negative - She had poor weight gain during the pregnancy - Prenatal ultrasounds revealed fetal growth restriction | | | | |
| **Past Medical/Surgical History** | | **Medications** | **Allergies** | **Family History** | | |
| None | | Prenatal vitamins | Amoxicillin (she gets a rash) | Mother has celiac disease | | |
| **Physical Examination** | | | | | |  |
| **General** | Anxious, agitated, asking inappropriate questions such as “What is going on?” and “Why are you touching me?”. Pt is wearing a hijab. If Oxygen placed, patient keeps trying to take it off. | | | |  |  |
| **HEENT** | Patent airway, normocephalic, atraumatic | | | |  |  |
| **Neck** | Supple, full ROM, tender goiter present | | | |  |  |
| **Lungs** | Tachypneic, increased work of breathing, speaking in one-word sentences, with crackles throughout | | | |  |  |
| **Cardiovascular** | Tachycardic with irregular rhythm. Brisk capillary refill, 2+ radial pulses | | | |  |  |
| **Abdomen** | Mild generalized tenderness, no hepatosplenomegaly, no masses, fundus firm at the level of the umbilicus | | | |  |  |
| **Neurological** | Normal and symmetric motor and sensory exam, able to follow commands | | | |  |  |
| **Skin** | Diaphoretic, warm | | | |  |  |
| **GU** | Normal GU exam with scant post-delivery bleeding | | | |  |  |
| **Psychiatric** | Agitated, anxious, repetitive | | | |  |  |

| Instructor Notes - Changes and CASE Branch Points | | |
| --- | --- | --- |
| **Intervention / Time point** | **Change in Case** | **Additional Information** |
| Patient placed on cardiopulmonary monitor; vital signs obtained, IV access obtained | If yes 🡪 | Vital signs displayed on monitor:  T 40°C  BP 110/75  HR 140  RR 24  O2 80% |
|  | If no 🡪 | RN can ask, “Would you like for me to put this patient on a monitor?” and/or “Would you like for me to obtain IV access?” |
|  | If still no 🡪 | RN says, “I’m going to put her on a monitor” and/or “I’m going to obtain IV access.” |
| Patient is placed on a nonrebreather mask | If yes 🡪 | Oxygen level increases to 92% |
|  | If no 🡪 | RN can ask, “Would you like for me to apply a nonrebreather mask?” |
|  | If still no 🡪 | RN says, “I’m going to apply a nonrebreather mask.” |
| Obtain pertinent history | If yes 🡪 | Continue the case |
|  | If no 🡪 | Partner/parent offers pertinent history |
| Obtain pertinent physical exam, including removal of hijab to reveal tender goiter | If yes 🡪 | Continue the case. Nurse will demonstrate findings. |
|  | If no 🡪 | RN fixes EKG leads and reveals that she noticed a tender neck mass. |
| Bloodwork is requested | If yes 🡪 | iSTAT venous gas available a few minutes after request.  CBC and BMP available several minutes after request.  Any other requested labs are sent to lab and pending.  See Appendix C |
|  | If no 🡪 | RN prompts, “Would you like for me to obtain any bloodwork?” |
| Chest X-ray (CXR) is requested | If yes 🡪 | CXR demonstrates pulmonary edema without cardiomegaly  See Appendix C |
|  | If no 🡪 | RN prompts, “Would you like for me to call for CXR?” |
| Electrocardiogram (EKG) is requested | If yes 🡪 | EKG demonstrates atrial fibrillation with rapid ventricular response  See Appendix C |
|  | If no 🡪 | RN prompts, “Would you like for me to perform an EKG?” |
| Point-of-care ultrasound (POCUS) of the heart, lungs, and IVC are requested | If yes 🡪 | POCUS demonstrates a hyperdynamic heart, pulmonary edema, and a plethoric IVC, respectively  See Appendix D, E, F |
|  | If no 🡪 | RN prompts, “The last time we had a critically ill patient, the physicians performed a POCUS of the heart, lungs, and IVC.” |
| Learners verbalize their interpretation of CXR, EKG, and POCUS results | If no 🡪 | RN asks what learners think of these tests, respectively. |
| 20 cc/kg normal saline (NS) IV bolus is requested and administered | If yes or if no 🡪 | Patient has no change in symptoms. Vitals remain unchanged. |
| Nitroglycerin or Lasix is requested | If yes 🡪 | Patient has no change in symptoms. Vitals remain unchanged. |
| BiPAP is applied | If yes 🡪 | Patient has no change in symptoms. RR decreases to 20, O2 sat increases to 95%. |
| Perform synchronized cardioversion for atrial fibrillation | If yes 🡪 | Patient converts to sinus tachycardia |
| Provide anticoagulation for atrial fibrillation | If yes 🡪 | Patient has no change in symptoms. Vitals remain unchanged. |
| Maternal fetal medicine, ObGyn, or endocrinology consulting service is called for guidance on management of thyroid storm in a post-partum patient | If yes 🡪 | Consultant recommends:   - Propylthiouracil (PTU) 300-1000mg PO or via NGT - Propranolol 0.5-2mg IV or 20-80mg PO/NG, OR esmolol 250 – 500 mcg/kg IV - Dexamethasone 2mg IV/IM OR Hydrocortisone 100mg IV^1^ - Holding Iodine for 1-2 hours |
|  | If no 🡪 | RN prompts, “Last time we had a post-partum patient, we called an MFM consult.” |
| Advised medications are given | If yes 🡪 | HR decreases from 140 🡪 110  RR decreases from 24 🡪 20  Patient appears less agitated |
|  | If no 🡪 | RN prompts, “Would you like for me to administer any medications?” |
| The patient is admitted and/or transferred to the intensive care unit | If yes 🡪 | The case concludes |
|  | If no 🡪 | RN prompts, “Did you already request a bed for this patient?” |

**Ideal Scenario Flow**

The learners enter the room to find the patient agitated, anxious, and short of breath. They immediately place the patient on bedside monitors, request IV access and send lab-work. They recognize tachycardia, tachypnea, hyperthermia, and hypoxia and place a nonrebreather mask which helps to improve oxygen saturations to the 90s. They perform a full physical exam and recognize a goiter and diaphoresis. An EKG and CXR are ordered, and a cardiac, thoracic, and IVC POCUS are performed, which reveal atrial fibrillation with rapid ventricular rate, pulmonary edema, and evidence of high output heart failure. They recognize thyroid storm in a post-partum patient, look up the appropriate initial medical management using a just-in-time resource, and initiate the appropriate treatment. They concurrently or subsequently consult the maternal-fetal medicine or another appropriate service, who recommends or confirms the appropriate treatment. The patient is administered the appropriate treatment and admitted or transferred to an intensive care unit.

Anticipated Management Mistakes

Specific prompts and responses for anticipated management mistakes are outlined in the “Instructor notes- changes and branch points” section above. To summarize, they include:

- Difficulty utilizing the standardized patient (SP). We found that some learners did not perform a physical exam on the SP because they felt uncomfortable doing so. We modified our sessions to include a very brief introduction to the utilization of an SP for simulation cases, including specific instructions to treat the SP like a real patient and perform any examination that they would perform in a true scenario, except for a pelvic exam.
- Failure to obtain pertinent post-partum history. If the learners do not obtain the salient points of the history that thyroid disease during pregnancy (poor weight gain, palpitations), then the simulated patient and/or family member can volunteer this information to the learners. Any history elements that the learners fail to obtain during the simulation should be discussed in the debriefing session.
- Failure to obtain pertinent physical exam finding of thyromegaly. If the learners do not elicit the pertinent exam finding of thyromegaly, then the confederate RN can prompt by exposing the neck while adjusting patient’s leads or placing leads for EKG placement and pointing out the neck mass to the learners. (Photo, found in Appendix C, can be passed around at this time). Any physical exam elements that the learners fail to identify during the simulation should be discussed in the debriefing session.
- Failure to recognize acute respiratory distress and hypoxia. If learners fail to recognize the patient’s respiratory distress and hypoxia, then the confederate RN should verbally bring attention to the patient’s distress and mention the hypoxia (“Did you see her O2 sats?”, “She is really working to breathe.”). If the learners fail to recognize these signs in a timely manner, then this should be discussed in the debriefing session.
- Uncertainty about treatment for postpartum thyroid storm. Many of our learners were unfamiliar with the treatment of post-partum thyroid storm. The confederate RN may prompt Obstetric or MFM consultation if needed (“Last night we had a post-partum patient and the doc called an OB for a consult.” “Can we admit her here? Do we have an OB floor?”). We created specific debriefing materials (Appendix H) to cover this information.
- Failure to consult OB, MFM, or endocrine. Specialist consultation will be very important in the ongoing management and disposition of this patient. If the learners do not request consultation, the confederate RN may suggest it (“Last night we had a post-partum patient and the doc called an OB for a consult” “Can we admit her here? Do we have an OB floor?”). This should also be discussed in the debriefing session after the simulation.

Reference

1. Foley MR ST, Garite TJ. *Obstetric Intensive Care Manual.* Fifth Edition ed: McGraw Hill; 2018.
