# Supplementary material for: Thyrotoxicosis in a Postpartum Adolescent: A Simulation Case for Emergency Medicine Providers
Source: MedEdPORTAL. 2020 Sep 10;16:10967. doi: 10.15766/mep_2374-8265.10967 (PMC7485909; doi:10.15766/mep_2374-8265.10967)
Supplement: Supplementary file 1 — Thyroid Storm Simulation Case.docxSimulation Scenario Environment Checklist.docxThyroid Storm Case Labs - CXR, EKG & Photo.docxThyroid Storm Cardiac POCUS.mp4Thyroid Storm Lung POCUS.mp4Thyroid Storm IVC POCUS.mp4Thyroid Storm Debriefing Guide.docxThyroid Storm Debrief.pptxThyroid Storm Case Survey.docx [file mep_2374-8265.10967-s001.zip › B. Simulation Scenario Environment Checklist.docx]

**Simulation Scenario Environment Checklist: Post-Partum Thyroid Storm**

This checklist is to be used prior to each simulation to ensure a high-fidelity simulation environment for participants with all necessary items to care for the patient. The resources, medications and equipment available should reflect what is actually available to participants in real practice in their clinical environment. Therefore, the following list reflects what is often found in emergency departments for care of critically ill patients. Please note that not all the resources, medications or equipment listed are necessary for this particular simulation case.

RESOURCES

☐Pediatric Advanced Life Support (PALS) reference cards

☐Broselow Tape

☐Institution Specific Resources (may or may not be pertinent and/or available at specific institution)

☐Pediatric Medication Book

☐Pediatric Intubation Checklist

STANDARDIZED PATIENT

☐Gown

☐Hijab or scarf

☐Sheet

PEDIATRIC SIMULATION EQUIPMENT

☐Gloves (All sizes available for participants)

☐Monitor (HR, RR, Oxygen saturation, NIBP, temperature and ETCO_2_ monitor

☐Heart Rate monitor leads, Oxygen saturation probe, Blood pressure cuff (mulitple sizes), ETCO_2_ cannula

☐Oxygen hook-up on wall or cylinder

☐Suction cannister, tubing, and yankauer tip

☐Stethoscopes (one for each provider if available)

☐Towels

☐Nonrebreathers, nasal cannulas, BVMs (multiple sizes)

☐Intubation Tray:

☐Miller blade sizes 2,3,4

☐Macintosh blade sizes 2,3,4

☐Cuffed Endotracheal tubes (ETTs) sizes 6.0, 6.5, 7.0, 7.5, 8.0

☐Adult and Pediatric Stylets

☐Adult and pediatric size end-tidal CO_2_ colorimeters

☐McGill forceps

☐Syringe

☐Tape

☐Nasopharyngeal and Oropharyngeal Airways (multiple sizes)

☐Video laryngoscopy device, stylet and handles (multiple sizes)

☐Nasogastric tubes (multiple sizes)

☐Intravenous (IV) Supplies:

☐IV/Angiocath, various sizes

☐Tourniquet

☐Syringes

☐IV pole and pump

☐IV tubing and filters

☐Pressure bags

☐Specimen tubes

☐Gauze and Tape

☐Sharps Container

☐Code cart with:

☐Defibrillator

☐Defibrillator pads (pediatric and adult size)

☐Backboard

☐Step Stool

☐Bedside Ultrasound Machine

MEDICATIONS

☐Acetaminophen ☐Hydrocortisone

☐Adenosine ☐Dexamethasone

☐Amiodarone ☐Propranolol

☐Atropine ☐Propylthiouracil (PTU)

☐Ceftriaxone ☐Methimazole

☐Epinephrine 1:10,000 ☐Potassium Iodide

☐Epinephrine 1:1,000 ☐Esmolol

☐Etomidate ☐Metoprolol

☐Fentanyl ☐Atenolol

☐Ketamine ☐Diltiazem

☐Lidocaine

☐Lorazepam

☐Midazolam

☐Morphine

☐Normal Saline/Lactated Ringers

☐Ibuprofen

☐Rocuronium

☐Succinylcholine

☐Vancomycin
