# Supplementary material for: Thyrotoxicosis in a Postpartum Adolescent: A Simulation Case for Emergency Medicine Providers
Source: MedEdPORTAL. 2020 Sep 10;16:10967. doi: 10.15766/mep_2374-8265.10967 (PMC7485909; doi:10.15766/mep_2374-8265.10967)
Supplement: Supplementary file 1 — Thyroid Storm Simulation Case.docxSimulation Scenario Environment Checklist.docxThyroid Storm Case Labs - CXR, EKG & Photo.docxThyroid Storm Cardiac POCUS.mp4Thyroid Storm Lung POCUS.mp4Thyroid Storm IVC POCUS.mp4Thyroid Storm Debriefing Guide.docxThyroid Storm Debrief.pptxThyroid Storm Case Survey.docx [file mep_2374-8265.10967-s001.zip › G. Thyroid Storm Debriefing Guide.docx]

Thyroid Storm Debriefing Guide

Debriefing, an interactive, bidirectional and reflective discussion, is the most important feature of simulation-based education.^1^ There are several methods used to conduct debriefing in healthcare simulation and any would provide an adequate educational reflection for the brief scenario in our session. The key to all debriefing methods is to provide a supportive environment to ensure psychological safety, use open-ended questions to encourage active participation, and make sure to address the learning objectives. The debriefing method outlined below provides a simple yet complete structure for efficient debriefing.

Each debriefing session started with a brief “Introduction” (or “Pre-Briefing”) during which the ground rules for the debriefing session are set for all participants. This encourages a respectful and safe learning environment for optimal education and participation. The rest of the debriefing took the typical multi-phase structure of a reactions phase, analysis phase and summary phase and were led using “debriefing with good judgment” approach.^2-5^ This approach is a reflective approach in which the instructor adopts a “stance of genuine curiosity” and inquires about the learner’s thoughts and actions. It also uses advocacy and inquiry to makes objective observations about the learner’s actions to provoke discussion. The “Emotional Experience” phase of debriefing aims to encourage learners to process their emotions surrounding the case itself and/or their individual performance in the case. This is done early in the debrief in the hopes of allowing learners to move past these emotions into the remainder of the debrief. The “Teamwork & Communication” phase focuses on examining team dynamics and how that affected patient care. This section also utilized some “plus/delta” discussion questions to encourage discussion about what went well and what could be improved upon. The “Medical Management” phase allows the team to review the facts of the case and solidify many of the cases learning objectives. Lastly, the “Wrap Up” phase encourages learners to highlight their take home points and gives facilitators a final opportunity to ensure that learning objectives have been covered.

| Debriefing Guide:  (Please refer to the Learning Objectives and Critical Actions to help guide discussion) | |  |
| --- | --- | --- |
| **Debriefing Phase** | Suggested questions/phrases | Case-Specific Discussion Points & Reference Materials |
| **Introduction** | We are now going to take the next 20 minutes to debrief. This part of our session is actually more important than the actual case. As a reminder, our ground rules are as follows:   - 1. This is meant to be a safe learning environment   2. Simulation is a formative, interdisciplinary learning environment - no one is being evaluated (except the simulation instructors because we constantly evaluate ourselves on how to provide better educational sessions).   3. We all agree to the basic assumption that everyone here is intelligent, well trained, and cares about doing their best.   4. Thus, we all agree to the highest standard of professional conduct and courtesy to our colleagues in this debrief and in any further discussions about this mock code. |  |
| **Emotional Experience** | - That was a challenging/stressful/difficult case. Let’s talk about what just happened. - What part of this scenario was particularly challenging? Why? | - Validate learner’s reactions and emotions - Invite thoughts from several learners |
| **Teamwork & Communication** | - Who were the members of the care team and how were roles established or assigned? | - Team leader – Identifies leader role, clear direction, team coordination, invites feedback, stands at foot of bed - Airway MD - Manage airway, prepare for advanced airway if necessary, stands at head of bed - Survey MD – Primary and secondary survey, communicates back to team leader, frequent reassessment - Bedside Nurse – Obtains access, administers medications, call backs to team leader - Medication Nurse – Draws up meds as ordered, call backs to team leader |
|  | - What elements of team communication went well? - What could be improved upon? - Did everyone know what the team leader was thinking? - Did everyone on the team feel empowered to speak up to offer suggestions or additions to patient care? | Find examples of/suggest examples of the following:   - Closed loop communication – receivers of information confirm to information to the sender   - *Ex. Team leader – “Jennifer, please place the patient on nasal cannula” ; Jennifer – “Placing the patient on nasal cannula”* - Directed call outs – using team members names or specific roles to ensure orders/assignments are not missed   - *Ex. “John, please obtain a manual blood pressure” ; “Team leader, I am having difficulty obtaining IV access.”* - Shared Mental Model with periodic case re-summarization to assure that all team members know the working diagnosis, management priorities and next steps for patient care.   - *Ex. “Given the patient’s respiratory distress and altered mental status, I am concerned about X. Our priorities should be placing the patient on oxygen and obtaining IV access.”* - Team members welcomed to participate in management decisions   - *Ex. “I see sinus tach on the monitor. Does everyone agree?”; “Dr. Smith, I am concerned that IV fluids might worsen her respiratory distress if she is in heart failure.”* |
| **Medical Management** | - What happened when you first entered the room? - What led to the recognition of respiratory distress and altered mental status? | - C, A, Bs should be quickly assessed - O_2_, monitors and IV ordered |
|  | - What was your initial differential diagnosis? | - Peripartum Cardiomyopathy, Pre-eclampsia, Pulmonary Embolism, Amniotic Fluid Embolism, Myocardial Infarction, Anxiety, Septic Shock, Thyroid Storm |
|  | - Why/when did you suspect thyroid storm? (Or why did you NOT suspect thyroid storm?) | - Vital sign abnormalities - Thyromegaly on physical exam |
|  | - After [____], what happened next? - After [____], how did the team respond? - I noticed you chose to [____], tell us about your thought process at that point. |  |
|  | - What resources did you mobilize (or could you have called upon) to manage this peripartum patient in the Emergency Department? | - Institution specific: OB, Maternal-Fetal Medicine, etc - How specifically do you obtain these consultations and/or resources at your institution? |
|  | - What is the management for a patient with post-partum thyroid storm? | - Propylthiouracil (PTU) 300-1000mg PO or via NGT - Propranolol 0.5-2mg IV or 20-80mg PO/NG, OR esmolol 250 – 500 mcg/kg IV - Dexamethasone 2mg IV/IM OR Hydrocortisone 100mg IV^6^ - Hold Iodine for 1-2 hours - Present Powerpoint slides (Appendix H) here to provide complete, but brief, review of medical knowledge |
| **Cultural Sensitivity** | - How did the patient’s hijab influence your ability to perform a full physical exam? - How can this, or other specific cultural considerations, pose challenges in trying to evaluate and manage the critically ill patient?” |  |
| **Wrap Up** | - What unanswered questions do you have about this case? - What is your take home point? (go around the room and have every learner state one) |  |

1. Sawyer T, Eppich W, Brett-Fleegler M, Grant V, Cheng A. More Than One Way to Debrief: A Critical Review of Healthcare Simulation Debriefing Methods. *Simul Healthc.* 2016;11:209-217.

2. Rudolph JW, Simon R, Dufresne RL, Raemer DB. There's no such thing as "nonjudgmental" debriefing: a theory and method for debriefing with good judgment. *Simul Healthc.* 2006;1:49-55.

3. Rudolph JW, Simon R, Raemer DB, Eppich WJ. Debriefing as formative assessment: closing performance gaps in medical education. *Acad Emerg Med.* 2008;15:1010-1016.

4. Gardner R. Introduction to debriefing. *Semin Perinatol.* 2013;37:166-174.

5. Hart D, McNeil MA, Griswold-Theodorson S, Bhatia K, Joing S. High fidelity case-based simulation debriefing: everything you need to know. *Acad Emerg Med.* 2012;19:E1084.

6. Foley MR ST, Garite TJ. *Obstetric Intensive Care Manual.* Fifth Edition ed: McGraw Hill; 2018.
