# Supplementary material for: Thyrotoxicosis in a Postpartum Adolescent: A Simulation Case for Emergency Medicine Providers
Source: MedEdPORTAL. 2020 Sep 10;16:10967. doi: 10.15766/mep_2374-8265.10967 (PMC7485909; doi:10.15766/mep_2374-8265.10967)
Supplement: Supplementary file 1 — Thyroid Storm Simulation Case.docxSimulation Scenario Environment Checklist.docxThyroid Storm Case Labs - CXR, EKG & Photo.docxThyroid Storm Cardiac POCUS.mp4Thyroid Storm Lung POCUS.mp4Thyroid Storm IVC POCUS.mp4Thyroid Storm Debriefing Guide.docxThyroid Storm Debrief.pptxThyroid Storm Case Survey.docx [file mep_2374-8265.10967-s001.zip › H. Thyroid Storm Debrief.pptx]

## Slide 1
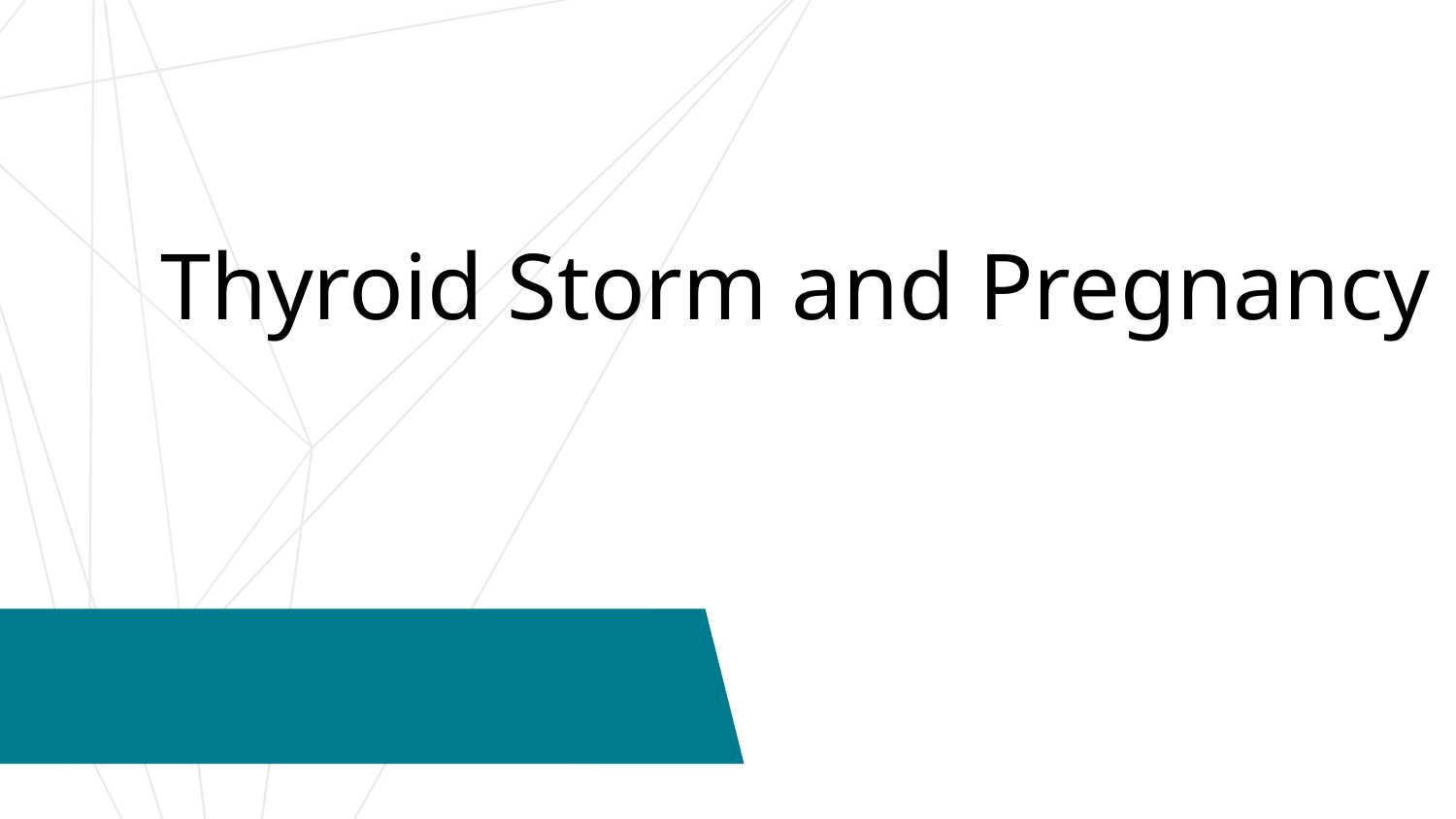

# Thyroid Storm and Pregnancy

## Slide 2
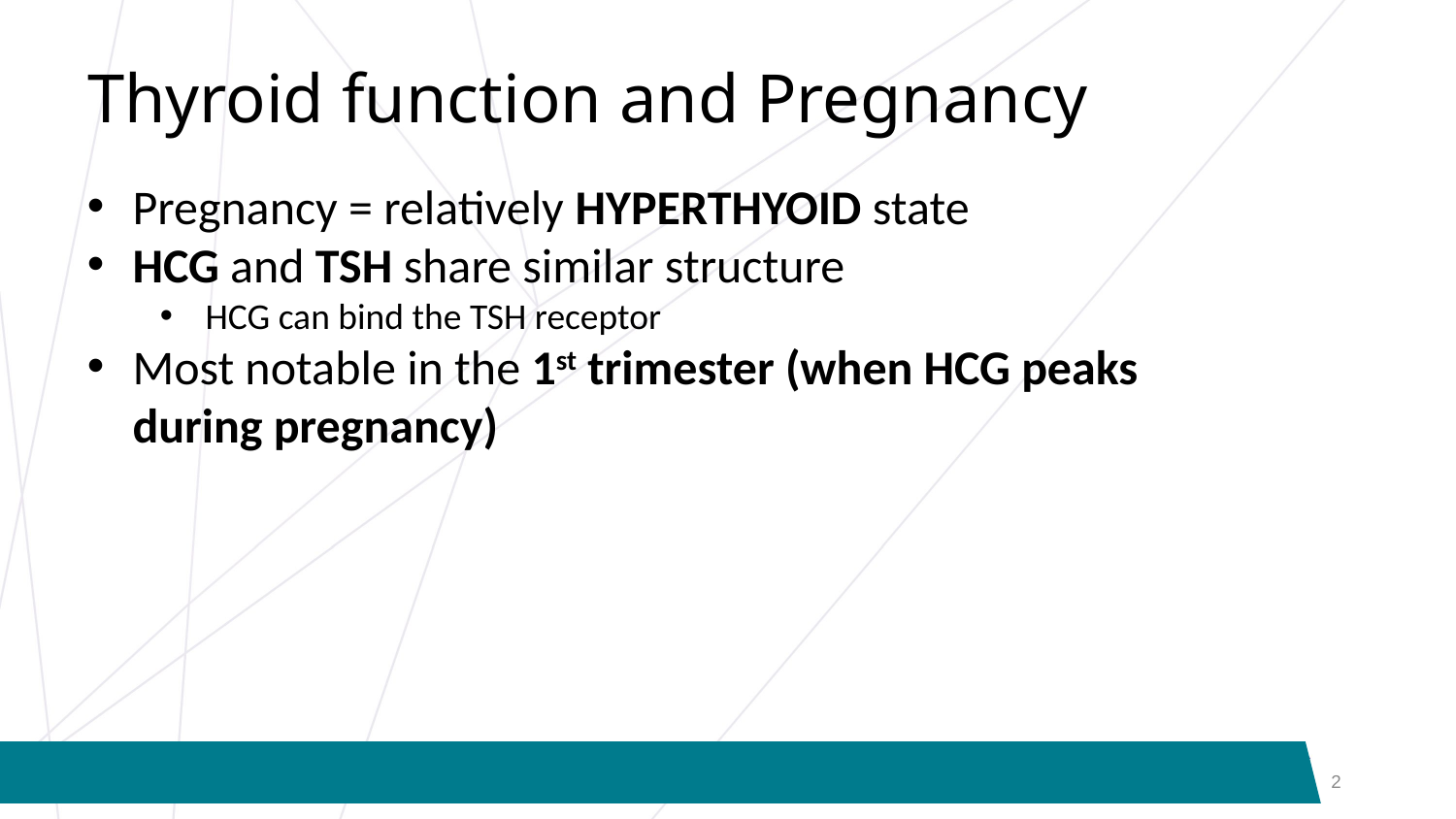

# Thyroid function and Pregnancy
Pregnancy = relatively HYPERTHYOID state
HCG and TSH share similar structure
HCG can bind the TSH receptor
Most notable in the 1st trimester (when HCG peaks during pregnancy)
1

## Slide 3
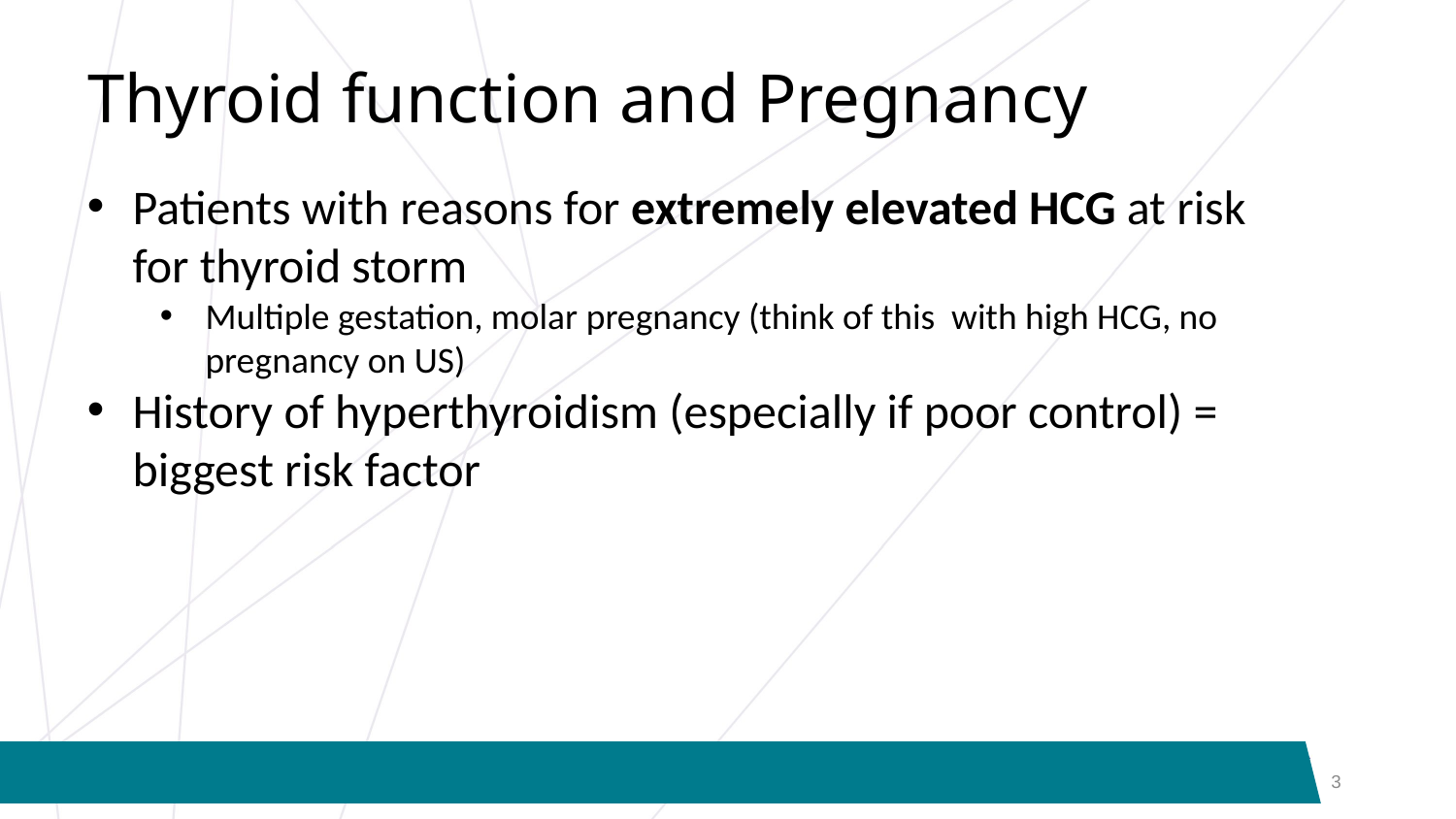

# Thyroid function and Pregnancy
Patients with reasons for extremely elevated HCG at risk for thyroid storm
Multiple gestation, molar pregnancy (think of this with high HCG, no pregnancy on US)
History of hyperthyroidism (especially if poor control) = biggest risk factor
2

## Slide 4
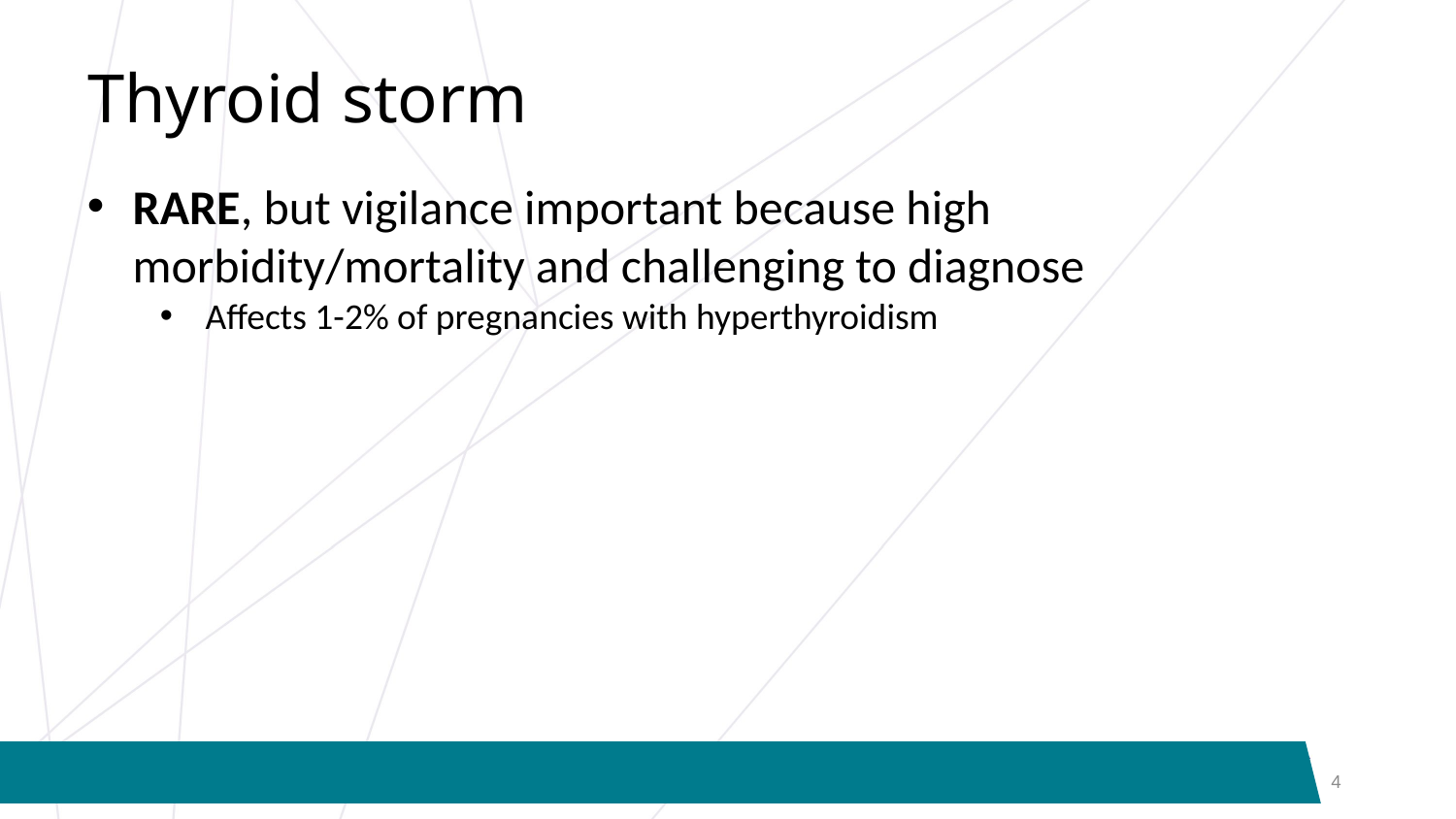

# Thyroid storm
RARE, but vigilance important because high morbidity/mortality and challenging to diagnose
Affects 1-2% of pregnancies with hyperthyroidism
3

## Slide 5
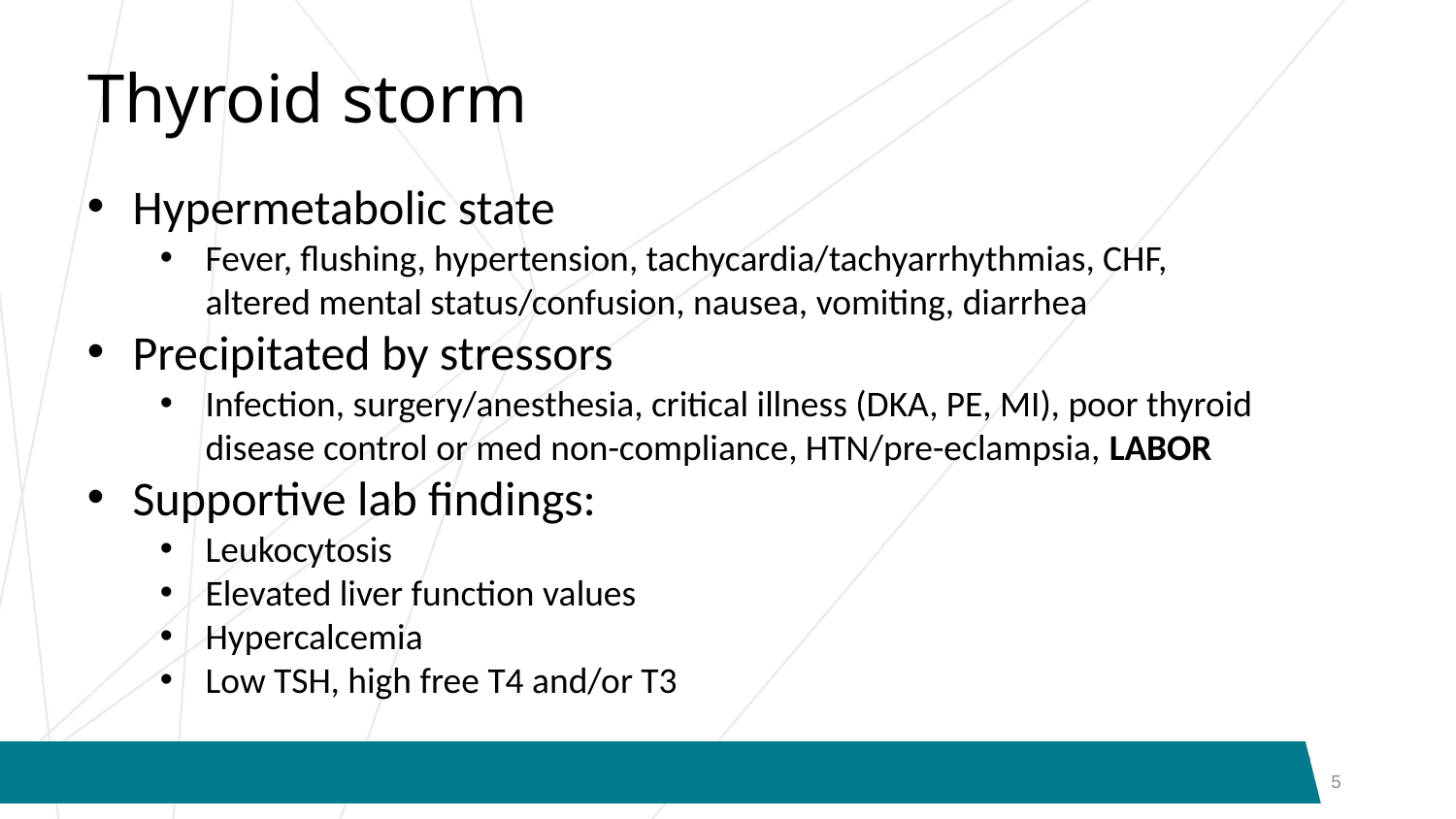

# Thyroid storm
Hypermetabolic state
Fever, flushing, hypertension, tachycardia/tachyarrhythmias, CHF, altered mental status/confusion, nausea, vomiting, diarrhea
Precipitated by stressors
Infection, surgery/anesthesia, critical illness (DKA, PE, MI), poor thyroid disease control or med non-compliance, HTN/pre-eclampsia, LABOR
Supportive lab findings:
Leukocytosis
Elevated liver function values
Hypercalcemia
Low TSH, high free T4 and/or T3
4

## Slide 6
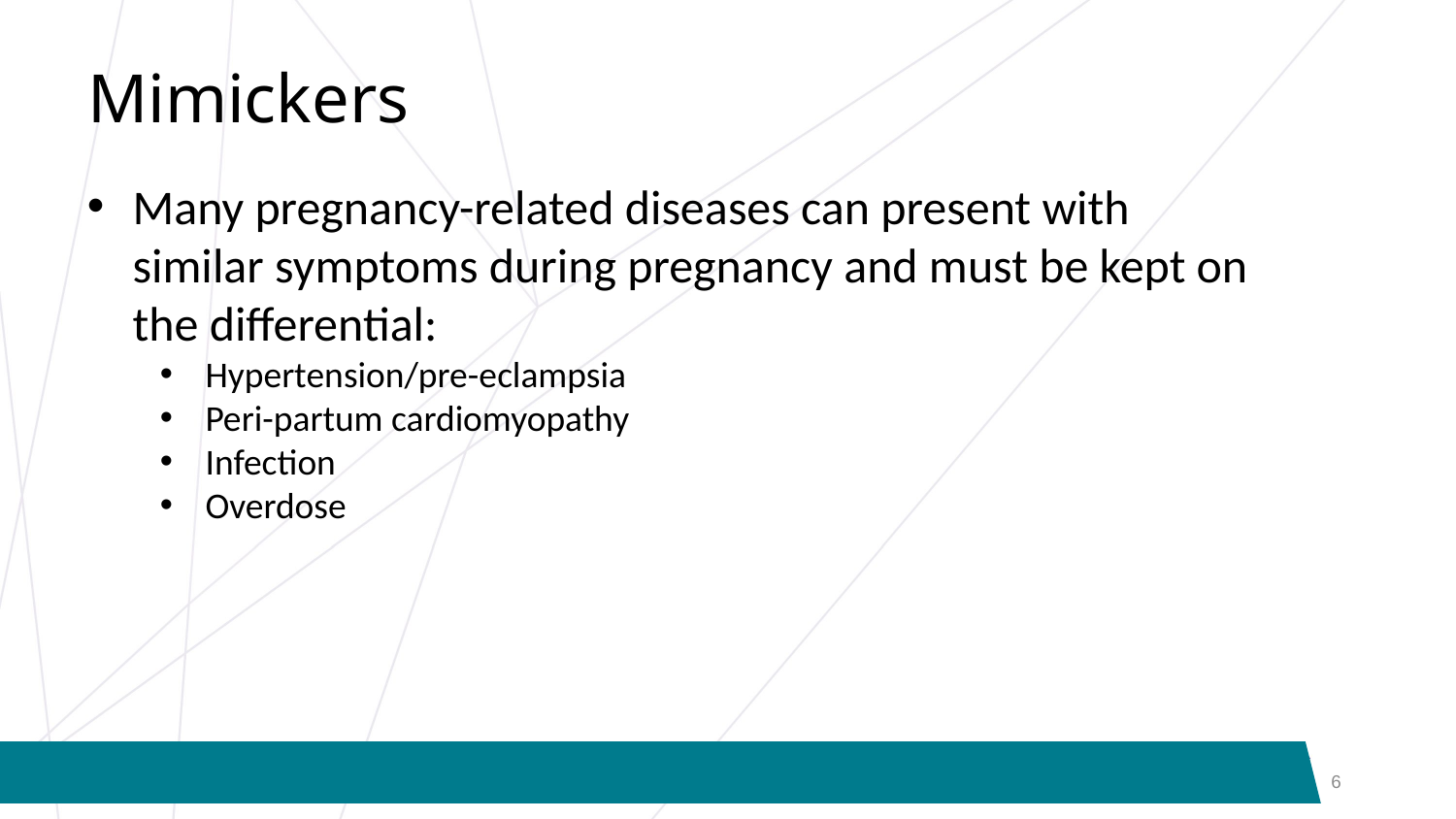

# Mimickers
Many pregnancy-related diseases can present with similar symptoms during pregnancy and must be kept on the differential:
Hypertension/pre-eclampsia
Peri-partum cardiomyopathy
Infection
Overdose
5

## Slide 7
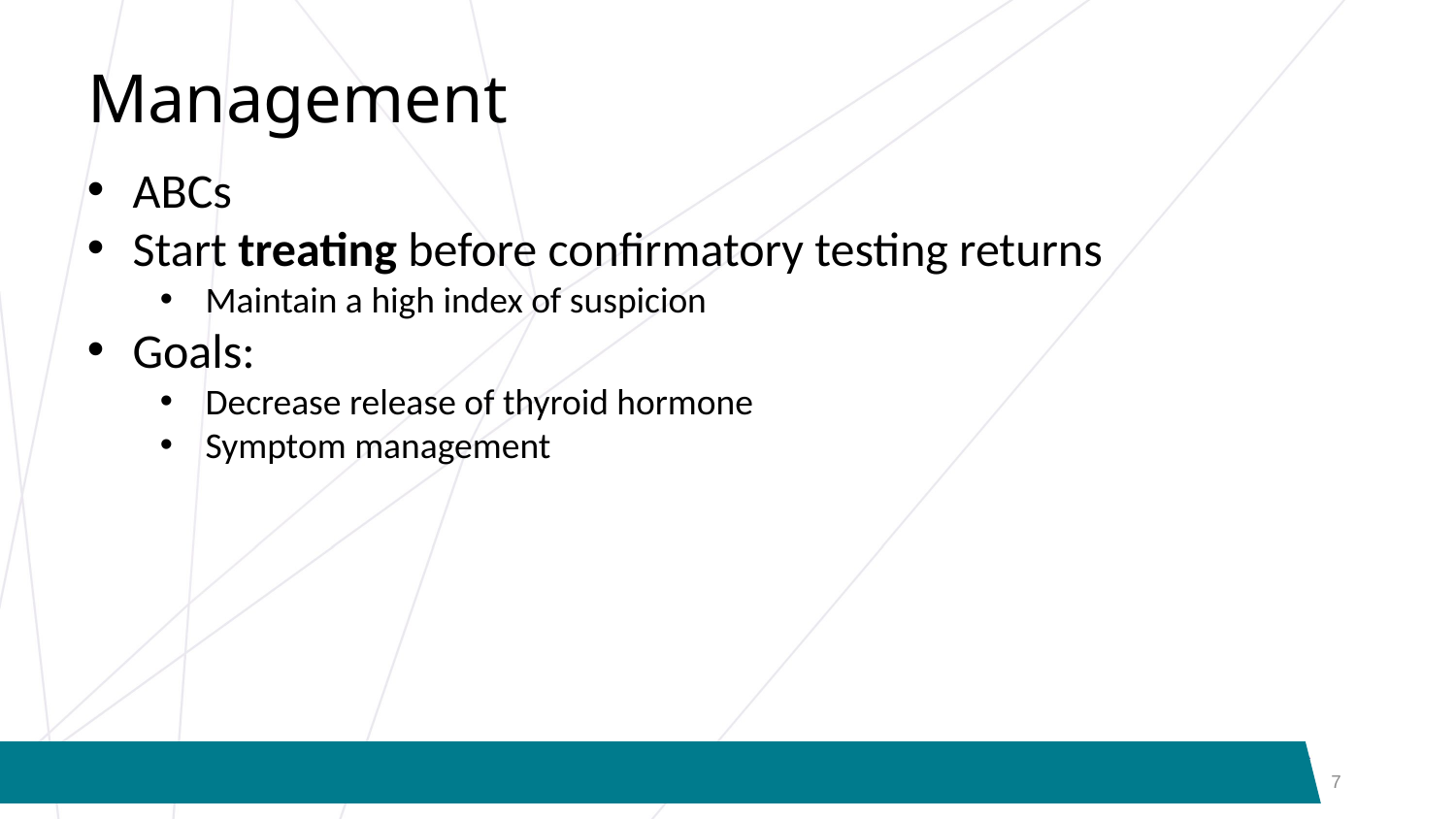

# Management
ABCs
Start treating before confirmatory testing returns
Maintain a high index of suspicion
Goals:
Decrease release of thyroid hormone
Symptom management
6

## Slide 8
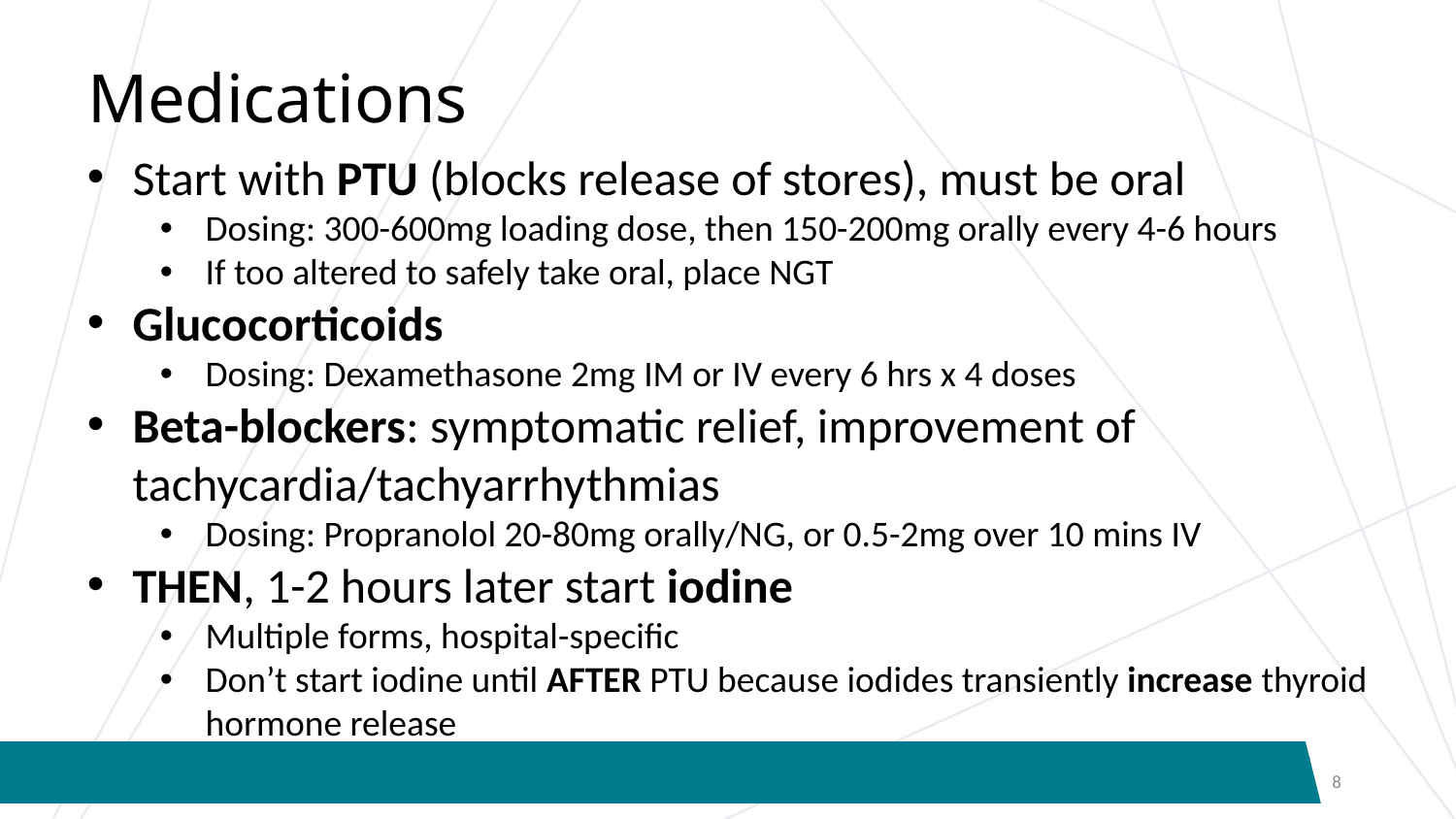

# Medications
Start with PTU (blocks release of stores), must be oral
Dosing: 300-600mg loading dose, then 150-200mg orally every 4-6 hours
If too altered to safely take oral, place NGT
Glucocorticoids
Dosing: Dexamethasone 2mg IM or IV every 6 hrs x 4 doses
Beta-blockers: symptomatic relief, improvement of tachycardia/tachyarrhythmias
Dosing: Propranolol 20-80mg orally/NG, or 0.5-2mg over 10 mins IV
THEN, 1-2 hours later start iodine
Multiple forms, hospital-specific
Don’t start iodine until AFTER PTU because iodides transiently increase thyroid hormone release
7

## Slide 9
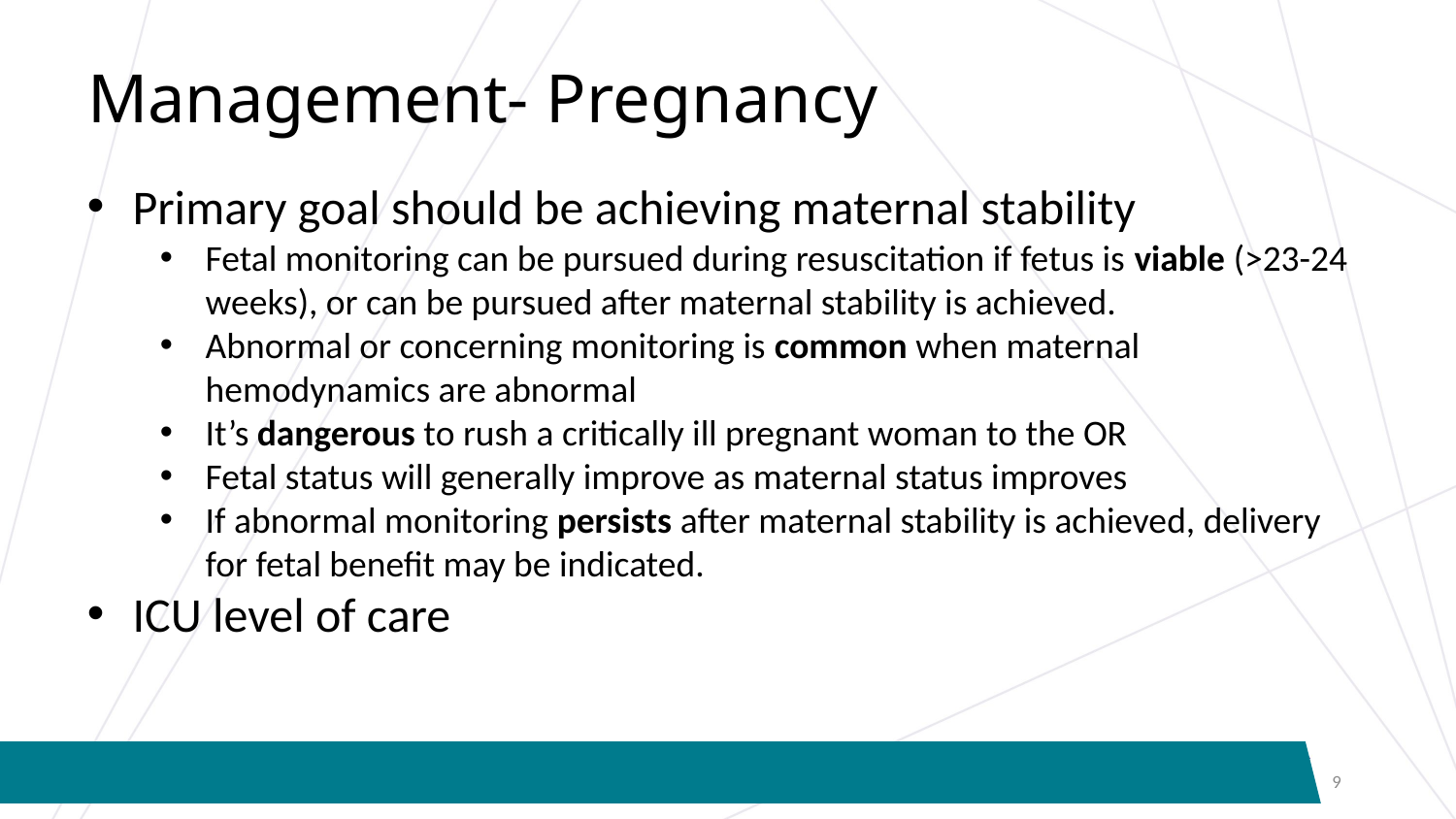

# Management- Pregnancy
Primary goal should be achieving maternal stability
Fetal monitoring can be pursued during resuscitation if fetus is viable (>23-24 weeks), or can be pursued after maternal stability is achieved.
Abnormal or concerning monitoring is common when maternal hemodynamics are abnormal
It’s dangerous to rush a critically ill pregnant woman to the OR
Fetal status will generally improve as maternal status improves
If abnormal monitoring persists after maternal stability is achieved, delivery for fetal benefit may be indicated.
ICU level of care
8

## Slide 10
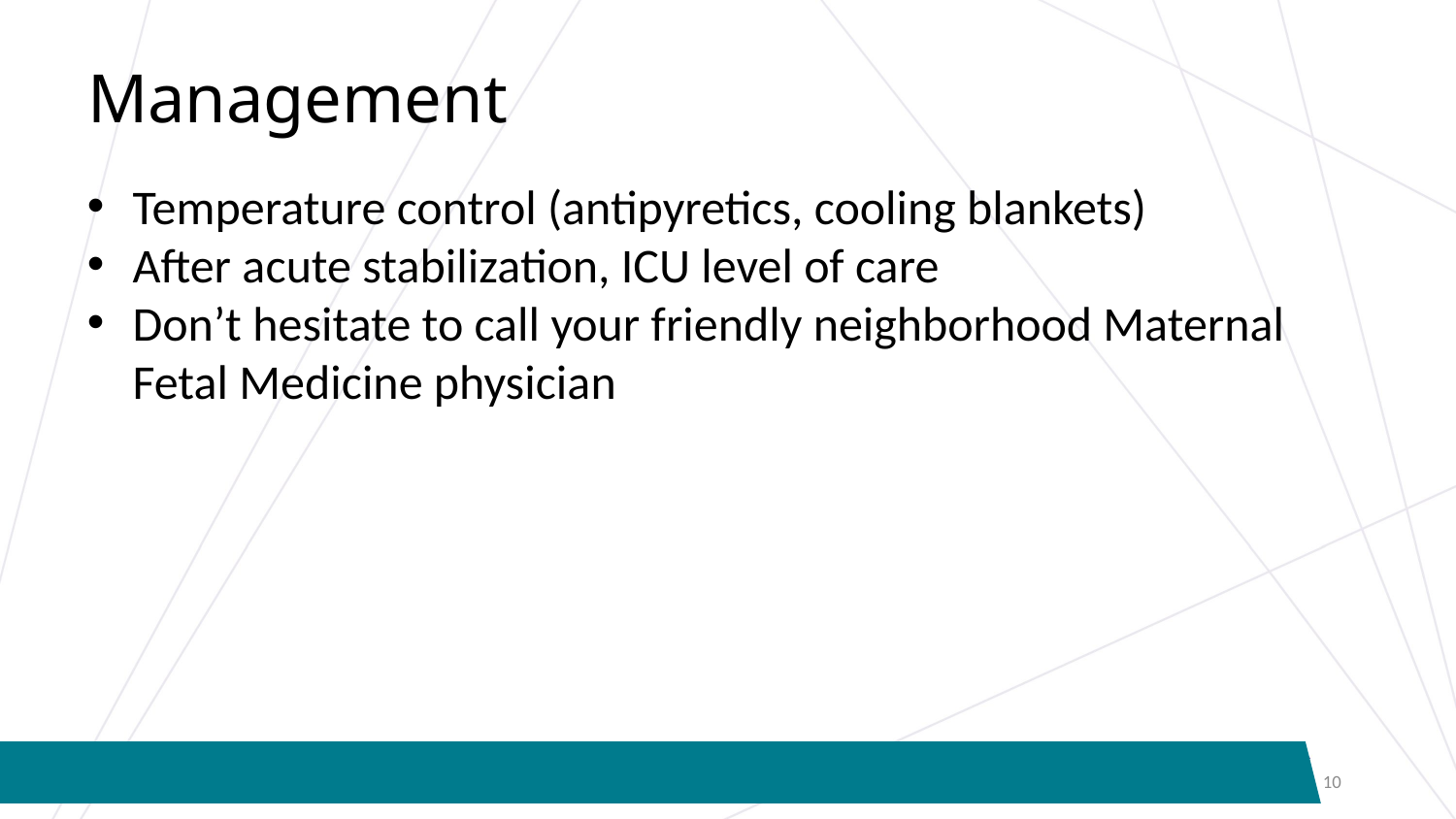

# Management
Temperature control (antipyretics, cooling blankets)
After acute stabilization, ICU level of care
Don’t hesitate to call your friendly neighborhood Maternal Fetal Medicine physician
9

## Slide 11
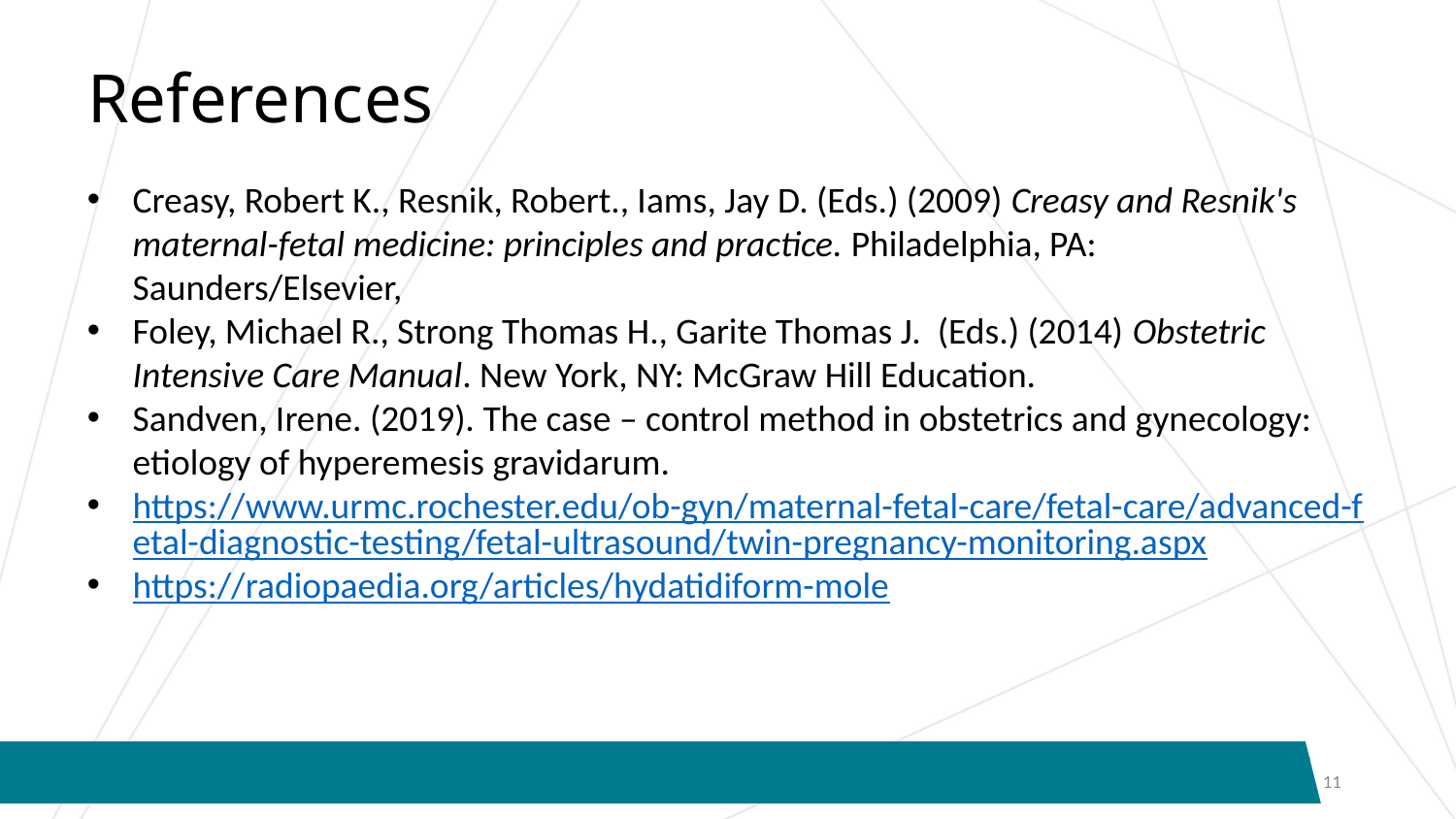

# References
Creasy, Robert K., Resnik, Robert., Iams, Jay D. (Eds.) (2009) Creasy and Resnik's maternal-fetal medicine: principles and practice. Philadelphia, PA: Saunders/Elsevier,
Foley, Michael R., Strong Thomas H., Garite Thomas J. (Eds.) (2014) Obstetric Intensive Care Manual. New York, NY: McGraw Hill Education.
Sandven, Irene. (2019). The case – control method in obstetrics and gynecology: etiology of hyperemesis gravidarum.
https://www.urmc.rochester.edu/ob-gyn/maternal-fetal-care/fetal-care/advanced-fetal-diagnostic-testing/fetal-ultrasound/twin-pregnancy-monitoring.aspx
https://radiopaedia.org/articles/hydatidiform-mole
10
