# Supplementary material for: Thyrotoxicosis in a Postpartum Adolescent: A Simulation Case for Emergency Medicine Providers
Source: MedEdPORTAL. 2020 Sep 10;16:10967. doi: 10.15766/mep_2374-8265.10967 (PMC7485909; doi:10.15766/mep_2374-8265.10967)
Supplement: Supplementary file 1 — Thyroid Storm Simulation Case.docxSimulation Scenario Environment Checklist.docxThyroid Storm Case Labs - CXR, EKG & Photo.docxThyroid Storm Cardiac POCUS.mp4Thyroid Storm Lung POCUS.mp4Thyroid Storm IVC POCUS.mp4Thyroid Storm Debriefing Guide.docxThyroid Storm Debrief.pptxThyroid Storm Case Survey.docx [file mep_2374-8265.10967-s001.zip › I. Thyroid Storm Case Survey.docx]

**Thyroid Storm Case Survey**

1. What is your current level of training?

☐ PGY 1 ☐ PGY 2 ☐ PGY 3 ☐ PGY 4

☐ PGY 5 ☐ PGY 6 ☐ PGY 7 ☐ Attending Physician

☐ Other _______________________

1. If you are a resident, what residency program are you in?

☐ Emergency Medicine ☐ OB/Gyn ☐ Other _______________________

1. Approximately, how many patients with thyroid storm have you cared for in your medical career? _____
2. Approximately, how many immediate post-partum patients have you cared for in the emergency setting? ____

**Please rate your agreement with the following statements:**

|  |  | Strongly Disagree | Disagree | Neither Agree nor Disagree | Agree | Strongly Agree |
| --- | --- | --- | --- | --- | --- | --- |
| 5. | This simulation case provided is relative to my work. | □ | □ | □ | □ | □ |
| 6. | This simulation case was realistic. | □ | □ | □ | □ | □ |
| 7. | This simulation case was effective in teaching thyroid storm management skills. | □ | □ | □ | □ | □ |
| 8. | The debrief created a safe environment. | □ | □ | □ | □ | □ |
| 9. | The debrief promoted reflection and team discussion. | □ | □ | □ | □ | □ |

**After participating in this session, how confident are you in your ability to:**

|  |  | Very Not confident | Not confident | Neutral | Confident | Very Confident |
| --- | --- | --- | --- | --- | --- | --- |
| 10 | Mobilize the appropriate personnel and resources to manage the peri-partum patient in the emergency department. | □ | □ | □ | □ | □ |
| 11 | Demonstrate recognition and management of respiratory distress and altered mental status in a post-partum patient. | □ | □ | □ | □ | □ |
| 12 | Evaluate for causes of respiratory distress and altered mental status in a post-partum patient. | □ | □ | □ | □ | □ |
| 13 | Recognize thyroid storm in a post-partum patient. | □ | □ | □ | □ | □ |
| 14 | Manage thyroid storm in a post-partum patient. | □ | □ | □ | □ | □ |

15. What did you take away from this case and/or how will it change your practice?

____________________________________________________________________

____________________________________________________________________

____________________________________________________________________

16. What specific changes would you make to improve this scenario?

____________________________________________________________________

____________________________________________________________________

17. Other comments or suggestions:

____________________________________________________________________

____________________________________________________________________

____________________________________________________________________

**Thank you for taking the time to complete this survey!**
