# Supplementary material for: Thyrotoxicosis in a Postpartum Adolescent: A Simulation Case for Emergency Medicine Providers
Source: MedEdPORTAL. 2020 Sep 10;16:10967. doi: 10.15766/mep_2374-8265.10967 (PMC7485909; doi:10.15766/mep_2374-8265.10967)
Supplement: Supplementary file 1 — Thyroid Storm Simulation Case.docxSimulation Scenario Environment Checklist.docxThyroid Storm Case Labs - CXR, EKG & Photo.docxThyroid Storm Cardiac POCUS.mp4Thyroid Storm Lung POCUS.mp4Thyroid Storm IVC POCUS.mp4Thyroid Storm Debriefing Guide.docxThyroid Storm Debrief.pptxThyroid Storm Case Survey.docx [file mep_2374-8265.10967-s001.zip › _Educational_Summary_Report_10967.pdf]

MedEdPORTAL® | The AAMC Journal of Teaching and Learning Resources

Original Publication

Open Access

Thyrotoxicosis in a Postpartum Adolescent: A Simulation Case for Emergency Medicine Providers

Julie I. Leviter, MD*, Sakina Sojar, MD, Nina K. Ayala, MD, Robyn Wing, MD, MPH

*Corresponding author: jleviter@gmail.com

Abstract

Introduction: Thyroid storm is a rare but life-threatening disease process that may be difficult to recognize and mimics other disease processes. It is critical for the emergency medicine clinician to be able to recognize thyroid storm in patients in order to effectively stabilize and treat them. **Methods:** In this standardized patient case, learners were faced with a 17-year-old postpartum woman presenting to the emergency department with respiratory distress and altered mental status secondary to thyroid storm. The target learners were emergency department providers, including residents, medical students, and advanced practice practitioners. Providers were expected to identify signs and symptoms of thyroid storm and to initiate appropriate diagnostic workup and management of this complex patient. Debriefing followed the simulation using a debriefing guide and PowerPoint presentation. **Results:** Thirty-four learners participated in this simulation. All learners agreed or strongly agreed that the simulation case was relevant to their work, and 97% agreed or strongly agreed that it was effective in teaching thyroid storm management skills. Eighty-five percent felt that following the simulation, they would be confident in their ability to recognize thyroid storm in a postpartum patient and to recognize and manage respiratory distress and altered mental status in a postpartum patient. **Discussion:** Learners felt that this case was effective in teaching the skills necessary for caring for postpartum patients with respiratory distress and altered mental status. Future directions include conducting the simulation in situ to include multidisciplinary teams and increasing the learner pool to include OB/GYN residents.

Keywords
Thyrotoxicosis, Emergency Medicine, Thyroid Storm, Atrial Fibrillation, Altered Mental Status, Pulmonary Edema, Congestive Heart Failure, Postpartum, Respiratory Distress

Educational Objectives

By the end of this session, learners will be able to:

1. Demonstrate recognition and management of respiratory distress and altered mental status in a postpartum patient.
2. Establish a differential diagnosis for respiratory distress and altered mental status in a postpartum patient.
3. Recognize the signs and symptoms of thyroid storm in a postpartum patient.
4. Initiate appropriate treatment and management of thyroid storm in a postpartum patient.
5. Mobilize the appropriate personnel and resources to manage the postpartum patient in the emergency department.
6. Demonstrate efficient and effective teamwork and communication skills.

Citation:
Leviter JI, Sojar S, Ayala NK, Wing R. Thyrotoxicosis in a postpartum adolescent: a simulation case for emergency medicine providers. *MedEdPORTAL*. 2020;16:10967.
https://doi.org/10.15766/mep_2374-8265.10967

Introduction

Thyroid storm is a rare but life-threatening disease process, with a mortality rate of 10%-30%.[1,2] The clinical presentation may mimic other entities and be difficult to recognize, particularly in a patient without a known history of thyroid disease. It is associated with numerous precipitants including trauma, burns, myocardial infarctions, pulmonary emboli, infections, or labor and delivery, particularly among those with undiagnosed and untreated hyperthyroidism.[1,3] The emergency provider may unwittingly focus exclusively on the primary precipitant, yet fail to recognize and manage a concurrent thyroid storm. Alternatively, when a postpartum patient presents with respiratory distress and heart failure, these may be mistaken for pulmonary embolism, amniotic

Copyright © 2020 Leviter et al. This is an open-access publication distributed under the terms of the Creative Commons Attribution license.

fluid embolism, pre-eclampsia, or peripartum cardiomyopathy. It is critical for the emergency medicine (EM) clinician to be able to recognize and promptly treat thyroid storm in a patient in order to avoid morbidity and death.

Thyroid storm is most commonly seen in women, and frequently in those with subacute, untreated, or poorly controlled hyperthyroidism.[1,2] While hyperthyroidism occurs in 0.1%-0.4% of pregnant women, commonly associated symptoms of tachycardia, hyperhidrosis, and anxiety can be mistaken for those of a normal pregnancy, and thus the diagnosis can remain elusive.[3,4] Congestive heart failure is reported to occur in up to 10% of patients presenting in thyrotoxicosis.[2,4] The physiologic stresses of labor and delivery can precipitate thyroid storm, which must be diagnosed and managed expeditiously based on clinical findings to reduce the risk of mortality.[1,2] Symptoms vary, but a high fever of 104°F to 106°F is usually present, as is significant tachycardia. Cardiac sequelae include palpitations, dyspnea, cardiac ischemia, and arrhythmias (particularly atrial fibrillation), which may progress to cardiovascular collapse and shock. Psychiatric manifestations include agitation, delirium, and confusion and may progress to stupor, obtundation, and coma. Gastrointestinal disturbances including vomiting, diarrhea, and liver dysfunction, and acute abdomen may occur as well. Treatment should begin as soon as thyroid storm is suspected. This includes propylthiouracil (PTU) to prevent new thyroid hormone production, as well as beta-blockers and corticosteroids to block the peripheral effects of thyroid hormone.[1,3] Additionally, supportive measures should be taken, including oxygen delivery and respiratory support, temperature control with antipyretics and cooling blankets, volume resuscitation as needed, and correction of electrolyte derangements.[3]

Simulation is a widely used educational tool that allows learners to develop skills and knowledge necessary for managing acutely ill patients in a safe learning environment.[5] Three prior *MedEdPORTAL* simulation cases feature patients with thyroid storm.[6-8] However, there are none, to our knowledge, with labor or delivery as the precipitant, nor are there any with respiratory distress as a prominent component of the presentation. Postpartum complications alone are an important topic for EM providers, and this simulation provides an opportunity to explore and discuss the differential diagnosis and available resources for an acutely ill patient in this population presenting to an emergency department.

Here, we offer a simulation case of an adolescent who presents with respiratory distress and altered mental status secondary to thyroid storm in the postpartum period. The case is targeted towards providers in pediatric EM (PEM) and EM. Both are groups that may have limited exposure to thyroid storm as well as to general postpartum complications, given their rarity in the emergency department setting. Learners are encouraged to develop a differential and management strategy for both respiratory distress and altered mental status in a postpartum patient, to mobilize the appropriate personnel and resources to manage the patient in the emergency department, and to manage thyroid storm in the patient. The case provides an opportunity to solidify trainee knowledge of the diagnosis and management of this rare but fatal disease entity in a safe and effective learning environment.

Methods

Development

This simulation case was developed by PEM physicians and a maternal-fetal medicine (MFM) physician to help learners recognize and manage thyroid storm in an adolescent postpartum patient. The case was developed for emergency department providers including EM residents, medical students, and advanced practice practitioners (APPs). It could also be run for PEM and EM attendings and fellows and adapted for OB/GYN and family medicine attendings, residents, and APPs. Prerequisite knowledge included identification and management of abnormal vital signs, abnormal physical exam findings, respiratory distress, altered mental status, and comprehension/interpretation of laboratory results and imaging.

Equipment/Environment

The setting for the case was a medical simulation center, which emulated a standard resuscitation bay in an emergency department. Vital signs were demonstrated on a monitor using standard LLEAP software by Laerdal. Available equipment and medications were listed in the simulation scenario environment checklist (Appendix B). Laboratory results and diagnostic modalities, including chest X-ray, electrocardiogram (EKG), and bedside thoracic and cardiac point-of-care ultrasound (POCUS) clips, were available to learners upon request (Appendices C-F).

Personnel

The personnel needed to implement this case included a simulation technician, a faculty instructor, and confederate actors or facilitators to play the roles of a nurse, the patient, and the patient's parent or partner (Appendix A). If limited confederates and/or actors are present, the role of the patient's parent or partner can be omitted. Two to four learners can participate in the case. Medical students were included in groups of residents as supporting providers and never served as the team leader.

Copyright © 2020 Leviter et al. This is an open-access publication distributed under the terms of the Creative Commons Attribution license.

The facilitators comprised a PEM attending, a PEM fellow, and an MFM fellow, the latter of whom served as the medical expert and delivered the presentation in Appendix H. The facilitators had conducted basic background research about thyroid storm and reviewed the presentation (Appendices G and H) prior to facilitating the simulation. Some specific challenges met during pilot testing are described in the Discussion section below.

Implementation

This simulation activity was implemented during scheduled EM didactics. There were 10-12 learners in each group. Four participants from the learner group actively participated in the simulation case at a time, while the remainder of the participants observed the simulation in the room. The simulated case ran for approximately 15 minutes, and an additional 20 minutes were devoted to debriefing.

At the start of the simulation, participants were informed that a postpartum adolescent had been taken into the resuscitation bay due to respiratory distress and altered mental status. The learners entered to see a patient, played by an actor, who was short of breath and asking inappropriate questions, as described in the simulation scenario (Appendix A). She was wearing a hijab, which covered her neck area. She was accompanied by a family member who was able to provide history. Participants were expected to instruct the nurse to place the patient on monitors, obtain IV access immediately, and recognize the vital sign derangements of tachycardia, tachypnea, hyperthermia, and hypoxemia. The patient's exam was significant for respiratory distress, rales on pulmonary auscultation, a goiter (revealed by a photo to learners if/when they examined the patient's neck under her hijab or after removing her hijab), agitation, and sweating.

Pulmonary findings, in conjunction with the patient's tachycardia and tachypnea, prompted lab work and imaging, including a chest X-ray, EKG, and cardiac and thoracic POCUS (Appendices C-F; clips in Appendices D-F are used with permission from Yale New Haven Department of Emergency Medicine). These demonstrated findings were concerning for pulmonary edema, atrial fibrillation with rapid ventricular response, a hyperdynamic heart, and high-output heart failure. Furthermore, history from the patient's partner or parent revealed palpitations, fatigue, hypertension, and poor weight gain during the pregnancy. The pertinent past medical history, in conjunction with the patient's goiter on exam, indicated to the learners that the patient suffered from hyperthyroidism.

Given the overall clinical picture, the learners had to recognize that this represented thyrotoxicosis and request to call a consulting service. Our case employed the use of an MFM fellow, but participants could request consultation from OB/GYN or endocrinology. The consultant spoke with the participants via a phone in the simulation center but could instead appear in person. The case ended when the participants ordered PTU, beta-blockers, and glucocorticoid for the patient as directed by the consultant (of note, they should not be prompted to order iodine as this would precipitate worsening of symptoms).

Debriefing

Directly following the simulation, facilitators led the debrief as described in the thyroid storm debriefing guide (Appendix G). Debriefing started with an introduction (or prebriefing) during which the ground rules for the session were set for all participants. This encouraged a respectful and safe learning environment for optimal education and participation. The rest of the debriefing took the typical multiphase structure (i.e., reactions phase, analysis phase, and summary phase) and was led using the debriefing-with-good-judgment approach with some advocacy and inquiry and plus-delta discussion questions.[9-12] During the analysis, or medical management phase, a PowerPoint presentation about hyperthyroidism and thyroid storm in the pregnant and postpartum patient (Appendix H) was delivered to the learners by an MFM physician. This educational PowerPoint could also be presented by the simulation facilitator. Lastly, the summary phase encouraged learners to highlight their take-home points and gave facilitators a final opportunity to ensure that learning objectives had been covered.

Assessment

Our session ended by asking participants to complete the thyroid storm case survey (Appendix I) in order to evaluate the effectiveness of the simulation in achieving the educational objectives as perceived by the participants. This survey, developed and edited by the authors of this simulation, used standard 5-point Likert-scale questions regarding educational objectives being met and participant confidence. The survey also invited participants to explain how the case might influence their future practice and how it could be improved for future learners.

Results

The case was run for three separate sessions involving 34 learners, including 24 EM residents, one physician assistant, six medical students, and three clinicians who did not specify their specific role within the department of emergency medicine.

After completing the simulation, all 34 learners completed the survey. All survey respondents either agreed or strongly agreed that the simulation case was relevant to their work, with an

Copyright © 2020 Leviter et al. This is an open-access publication distributed under the terms of the Creative Commons Attribution license.

average score of 4.8 on a 5-point Likert scale (1 = *strongly disagree,* 5 = *strongly agree*), and 97% agreed or strongly agreed that the simulation was effective in teaching thyroid storm management skills, with an average score of 4.7 on the same Likert scale (see Table 1). Eighty-five percent felt that following the simulation, they would be confident in their ability to (1) recognize thyroid storm in a postpartum patient, with an average score of 3.9 on a 5-point Likert scale (1 = *very unconfident,* 5 = *very confident*), and (2) recognize and manage respiratory distress and altered mental status in a postpartum patient, with an average score of 4.1 on the same Likert scale (see Table 2).

Table 2. Participant Assessment of Self-Confidence Related to Ability to Carry out Stated Learning Objectives ($N = 34$)

Statement	M^a
After participating in this session, how confident are you in your ability to:	
Mobilize the appropriate personnel and resources to manage the peripartum patient in the emergency department.	4.0
Demonstrate recognition and management of respiratory distress and altered mental status in a postpartum patient.	4.1
Evaluate for causes of respiratory distress and altered mental status in a postpartum patient.	3.9
Recognize thyroid storm in a postpartum patient.	3.9
Manage thyroid storm in a postpartum patient.	3.9

[a]Rated on 5-point Likert scale (1 = *very unconfident,* 3 = *neutral,* 5 = *very confident*).

Comments from the postsimulation evaluation form included the following:

- "I like this case because it keeps a rare diagnosis on my differential for postpartum patients."
- "Good reminder to fully expose the patient and perform complete physical exam."
- "Changed my practice by keeping a low threshold to involve OB/MFM in care of sick postpartum patients."
- "Really like that the MFM was present and interactive in this case. It really tied together all the major players and their thought process on the case."

Discussion

This simulation was intended to teach EM clinicians to recognize and manage thyroid storm in a postpartum patient effectively in a safe learning environment. This is a rare disease process with a high mortality; thus, it is important to build the skills to care for such patients. The learners in this simulation appreciated the review of thyroid storm recognition and management and found the case valuable to their education and training. The simulation also allowed for discussion about special considerations and physiology when caring for the peripartum and postpartum patient in the emergency department.

Simulated cases of thyrotoxicosis exist in the current literature; however, to our knowledge, our simulation is the first with labor or delivery as the precipitant of the thyroid storm. The case was developed with valuable input from MFM providers specializing in such medical complications of the peripartum period, which helped to make the scenario more realistic and the debriefing more comprehensive.

Table 1. Simulation Survey Results ($N = 34$)

Statement	M^a
This simulation case provided is relevant to my work.	4.8
This simulation case was realistic.	4.6
This simulation case was effective in teaching thyroid storm management skills.	4.7
The debrief created a safe environment.	4.9
The debrief promoted reflection and team discussion.	4.8

[a]Rated on 5-point Likert scale (1 = *strongly disagree,* 3 = *neither agree nor disagree,* 5 = *strongly agree*).

Several variations of this case are possible, depending on the resources available at the institution carrying out the simulation. With the availability of an obstetric trainer, the facilitators might decide to include delivery of an infant as part of the simulation, with thyroid storm physiology manifesting during or directly after the delivery. This would provide an added skill training for EM providers, who would practice delivery and neonatal resuscitation, as well as add complexity to the case that may be desired. Another variation would be to use a high-fidelity mannequin as opposed to a standardized patient (SP). This would have the benefit of being able to recreate exam findings (e.g., pulmonary rales, tachycardia with irregular rhythm, etc.) such that the learners could practice gathering physical exam information from the SP herself. However, it is difficult to adequately portray all aspects of an agitated patient, such as gestures, eye contact, and subtle movements, using a mannequin.

There are a few limitations to this simulation that resulted from the utilization of an SP. Careful scripting and instructions to the SP were necessary for the case to proceed. For example, the SP had to act out altered mental status with agitation and anxiety. We found that specific instructions, such as directing the SP to repeatedly pull off her oxygen mask and ask repetitive questions, as detailed in Appendix A, were helpful. Another limitation of this simulation was the physical exam finding of a goiter, which was challenging to replicate. In our case, we used a hijab to cover the patient's neck and showed participants a photo of a goiter when they successfully examined the patient's neck. This enforced the importance of performing a complete physical exam, as well as bringing up a discussion in the debrief about cultural sensitivity. In future simulations, any article of clothing could be used for neck coverage, including a scarf or turtleneck sweater. Alternatively,

Copyright © 2020 Leviter et al. This is an open-access publication distributed under the terms of the Creative Commons Attribution license.

if facilitators have the appropriate moulage, an SP might be able to have a goiter on exam, which would be the highest level of fidelity, although more resource intensive.

Another limitation of this case was that the consultant was readily available to provide guidance on the management of this critically ill patient. One alternative option would be to delay the consultant's response until the learners have used a just-in-time resource to initiate appropriate management. After doing so, the consultant could then be present to give additional advice and redirect the team as needed.

The utilization of POCUS in simulation was important because most EM providers now use POCUS as an examination and diagnostic tool. Since our SP obviously did not have abnormal POCUS findings, we played video clips of corresponding ultrasound clips if/when the participants verbalized that they would perform a particular POCUS exam. An institution using mannequins equipped with simulation technology, rather than an SP, could allow participants to practice their POCUS skills as part of the simulation and broaden their learning objectives.

Overall, this simulation case ran smoothly and was well received by learners at our institution. EM providers are required to maintain a broad differential diagnosis, including rare and potentially fatal etiologies of clinical decompensation. They are responsible for considering special circumstances in certain populations (e.g., the pediatric patient and the postpartum patient) in creating those differential diagnoses. They often are faced with the task of stabilizing a patient while not knowing what the primary disease process may be. In addition to accomplishing the specific learning objectives for this exercise, this simulation case highlights the overall listed challenges of the emergency department clinician, making it a valuable learning experience.

Future directions for utilization and study of this simulation include conducting it in the emergency department setting. An in situ simulation would allow for multidisciplinary education, assist in determining accessibility and availability of equipment and medications, and permit true testing of systems such as mobilizing consultation services. Additionally, the simulation could also be performed with family medicine or OB/GYN providers as participants.

Appendices

A. Thyroid Storm Simulation Case.docx

B. Simulation Scenario Environment Checklist.docx

C. Thyroid Storm Case Labs - CXR, EKG & Photo.docx

D. Thyroid Storm Cardiac POCUS.mp4

E. Thyroid Storm Lung POCUS.mp4

F. Thyroid Storm IVC POCUS.mp4

G. Thyroid Storm Debriefing Guide.docx

H. Thyroid Storm Debrief.pptx

I. Thyroid Storm Case Survey.docx

All appendices are peer reviewed as integral parts of the Original Publication.

Julie I. Leviter, MD: Assistant Professor of Clinical Pediatrics, Department of Pediatric Emergency Medicine, Yale University School of Medicine; ORCID: https://orcid.org/0000-0001-5770-8796

Sakina Sojar, MD: Pediatric Emergency Medicine Fellow, Departments of Emergency Medicine and Pediatrics, Warren Alpert Medical School of Brown University and Hasbro Children's Hospital/Rhode Island Hospital; ORCID: https://orcid.org/0000-0002-2977-8479

Nina K. Ayala, MD: Maternal Fetal Medicine Fellow, Department of Obstetrics and Gynecology, Warren Alpert Medical School of Brown University and Women & Infants Hospital of Rhode Island

Robyn Wing, MD, MPH: Assistant Professor, Departments of Emergency Medicine and Pediatrics, Division of Pediatric Emergency Medicine, Warren Alpert Medical School of Brown University and Rhode Island Hospital/Hasbro Children's Hospital; Director of Pediatric Simulation, Lifespan Medical Simulation Center

Disclosures
None to report.

Funding/Support
None to report.

Ethical Approval
Reported as not applicable.

References

1. Chiha M, Samarasinghe S, Kabaker AS. Thyroid storm: an updated review. *J Intensive Care Med*. 2015;30(3):131-140. https://doi.org/10.1177/0885066613498053
2. Kitazawa C, Aoki S, Takahashi T, Hirahara F. Acute respiratory failure due to thyroid storm developing immediately after delivery. *Clin Case Rep*. 2015;3(12):997-999. https://doi.org/10.1002/ccr3.422
3. Ma Y, Li H, Liu J, Lin X, Liu H. Impending thyroid storm in a pregnant woman with undiagnosed hyperthyroidism: a case

Copyright © 2020 Leviter et al. This is an open-access publication distributed under the terms of the Creative Commons Attribution license.

report and literature review. *Medicine (Baltimore)*. 2018;97(3):e9606. https://doi.org/10.1097/MD.0000000000009606

4. Mestman JH. Hyperthyroidism in pregnancy. *Clin Obstet Gynecol*. 1997;40(1):45-64. https://doi.org/10.1097/00003081-199703000-00007
5. Weinberg ER, Auerbach MA, Shah NB. The use of simulation for pediatric training and assessment. *Curr Opin Pediatr*. 2009;21(3):282-287. https://doi.org/10.1097/MOP.0b013e32832b32dc
6. Rader K, Morrison R. Thyroid storm presenting as gastroenteritis. *MedEdPORTAL*. 2011;7:8436. https://doi.org/10.15766/mep_2374-8265.8436
7. Bhatia K. Thyroid storm presenting as new-onset psychosis. *MedEdPORTAL*. 2014;10:9735. https://doi.org/10.15766/mep_2374-8265.9735
8. Burns R, Stone K, Reid J, Malik F, Cheng A. Pediatric Emergency Medicine Simulation Curriculum: thyroid storm. *MedEdPORTAL*. 2015;11:10062. https://doi.org/10.15766/mep_2374-8265.10062
9. Hart D, McNeil MA, Griswold-Theodorson S, Bhatia K, Joing S. High fidelity case-based simulation debriefing: everything you need to know. *Acad Emerg Med*. 2012;19(9):E1084. https://doi.org/10.1111/j.1553-2712.2012.01423.x
10. Rudolph JW, Simon R, Dufresne RL, Raemer DB. There's no such thing as "nonjudgmental" debriefing: a theory and method for debriefing with good judgment. *Simul Healthc*. 2006;1(1):49-55. https://doi.org/10.1097/01266021-200600110-00006
11. Rudolph JW, Simon R, Raemer DB, Eppich WJ. Debriefing as formative assessment: closing performance gaps in medical education. *Acad Emerg Med*. 2008;15(11):1010-1016. https://doi.org/10.1111/j.1553-2712.2008.00248.x
12. Gardner R. Introduction to debriefing. *Semin Perinatol*. 2013;37(3):166-174. https://doi.org/10.1053/j.semperi.2013.02.008

Received: September 26, 2019
Accepted: February 29, 2020
Published: September 10, 2020

Copyright © 2020 Leviter et al. This is an open-access publication distributed under the terms of the Creative Commons Attribution license.
